# Melioidosis Presenting as Parapharyngeal Abscess

**DOI:** 10.31662/jmaj.2025-0546

**Published:** 2026-02-13

**Authors:** Srujana Mohanty, Rana Pratap Dutta, Pradeep Pradhan

**Affiliations:** 1 Department of Microbiology, All India Institute of Medical Sciences, Bhubaneswar, Odisha, India; 2 Department of ENT, All India Institute of Medical Sciences, Bhubaneswar, Odisha, India

**Keywords:** abscess, *Burkholderia pseudomallei*, diabetes mellitus, melioidosis, parapharyngeal abscess

## To the Editor

We read with interest the article by Wang et al. ^(1)^ entitled “Melioidosis presenting as retropharyngeal abscess” in the October 2025 issue of *JMA Journal*. The authors have described an interesting and rare case of melioidosis deep neck space infection (DNSI) caused by *Burkholderia pseudomallei* presenting as a retropharyngeal abscess. We wish to share our experience of a similar case of melioidosis DNSI presenting as a parapharyngeal abscess to raise awareness about this rare manifestation of *B. pseudomallei* infection, as both the entities (melioidosis and DNSI), though serious and life-threatening in nature, are potentially treatable ^(2)^.

**Figure 1.**
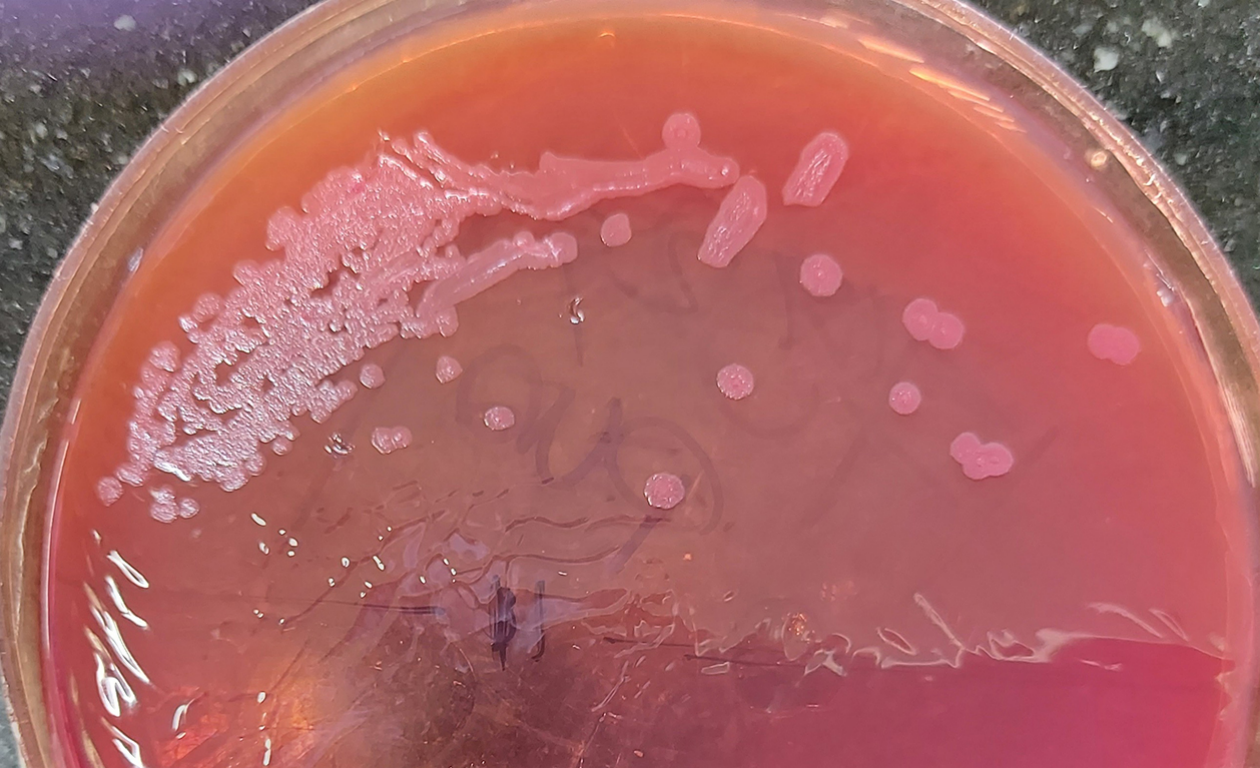
Non-lactose fermenting, oxidase-positive, wrinkled colonies of *Burkholderia pseudomallei.*

A 31-year-old man, a farmer by profession and a known diabetic, presented with a 10-day history of an increasingly painful swelling over the right side of his neck, associated with fever and severe weakness. The patient was unable to open his mouth and had difficulty swallowing food. He had sustained an injury on the right side of his neck while carrying wooden logs, following which he had developed a non-healing ulcer at the site, leading to a swelling, which gradually extended to the base of the mouth and surrounding areas. Clinical examination showed a visible swelling over the right sternocleidomastoid muscle extending to the submandibular region. A diagnosis of parapharyngeal abscess was made based on the clinical examination, together with radiological imaging findings. Blood chemistry suggested an extremely poor diabetic control with a fasting blood sugar level of 305 mg/dL, postprandial sugar 192 mg/dL, and glycated hemoglobin level of 12.6%. Following an incision and drainage, about 20-30 mL of thick, purulent pus was submitted for culture, which yielded growth of a single type of non-lactose fermenting, oxidase-positive, wrinkled colonies identified as *B. pseudomallei* by staining characteristics (gram-negative rod with safety-pin appearance) and VITEK-2 (Figure 1). The isolate was found to be susceptible to antibiotics (ceftazidime, trimethoprim-sulfamethoxazole, imipenem, amoxicillin-clavulanate, and doxycycline) with minimum inhibitory concentration noted (in μg/mL) as: 1.5, 2, 0.75, 4, and 0.5, respectively. Following treatment with intravenous ceftazidime 2g every 8 hours for 3 weeks, with resolution of the abscess and decrease in systemic symptoms, he was discharged with advice of oral trimethoprim-sulfamethoxazole for eradication therapy.

Melioidosis is currently showing a surge in locations other than its classically described endemic zones ^(3), (4), (5)^. The patient had several predisposing factors for *B. pseudomallei* acquisition, such as contact with soil, traumatic injury, and underlying diabetes. Clinicians in tropical zones should increasingly keep in mind the possibility of melioidosis in acute pyogenic infections, including DNSIs.

## Article Information

### Author Contributions

Provided substantial contribution to the concept and design of the study; acquisition, analysis, and interpretation of data, did the literature search, and revised the work for important intellectual content. She is the Corresponding author who gave the final approval for the manuscript to be published: Srujana Mohanty. Provided substantial contribution to the acquisition, analysis, and interpretation of data for the work, did the literature search, and edited the manuscript: Rana Pratap Dutta. Was the treating physician and contributed to the acquisition, analysis, and interpretation of data for the study as well as critically revised the work for important intellectual content: Pradeep Pradhan.

### Conflicts of Interest

None

### Consent Statement

Written consent has been obtained from the patient to publish the information, including the photographs

### Data Availability Statement

Data sharing is not applicable to this article as no new data were created or analyzed in this study
